# Reviewer Acknowledgements

**DOI:** 10.1186/1471-2296-14-18

**Published:** 2013-02-27

**Authors:** Christopher Foote

**Affiliations:** 1 BMC Series, BioMed Central, 236 Grays Inn Road, London, WC1X 8HB, UK

## Abstract

**Contributing reviewers:**

The editors of BMC Family Practice would like to thank all the reviewers who have contributed to the journal in volume 13 (2012).

## 

Harry Ahmed

UK

Raymond Anderson

USA

Claire Anderson

UK

Bengt Arnetz

USA

Benoit Arsenault

Netherlands

Özgür Arun

Turkey

Joseph Azuri

Israel

Caroline A Baan

Netherlands

Maureen Baker

UK

Richard Baker

UK

Arun Baksi

UK

Bijal Balasubramanian

USA

Elizabeth Balk

USA

Alan Barnard

South Africa

Melanie Barwick

Canada

Joanna Bates

Canada

Erika Baum

Germany

Justin Beilby

Australia

Jim Bellows

USA

Abdulbari Bener

Qatar

Kathleen Bennett

Ireland

Annette Berendsen

Netherlands

Alan Bittles

Australia

Laura Black

USA

Patricia Blackburn

Canada

Mitchel Blair

UK

Alison Blenkinsopp

UK

Pauline Boeckxstaens

Belgium

Marieke Bolkenbaas

Netherlands

Katrien Bombeke

Belgium

Stefan Bösner

Germany

Alex Boussioutas

Australia

Colin Bradley

Ireland

Bonnie Braun

USA

Angela Brega

USA

Eileen Britt

New Zealand

Helena Britt

Australia

Fiona Brooks

UK

Lauro Bucchi

Italy

Thomas Buckley

Australia

Olivier Bugnon

Switzerland

Teresa Burgess

Australia

Christopher Burton

UK

Ligaya Butalid

Netherlands

Molly Byrne

Ireland

David Cade

UK

Maria Caiata Zufferey

Switzerland

Ian Cameron

Australia

Josip Car

UK

Adrian Carter

Australia

Gillian Caughey

Australia

Venija Cerovecki

Croatia

Carolyn Chew-Graham

UK

Stefano Ciatto

Italy

Alexander Clark

Canada

John Cleland

UK

Sarah Clement

UK

Orit Cohen Castel

Israel

Judith Cole

UK

Beverly Collett

UK

Claire Collins

Ireland

Andrew Coogan

Ireland

Ian Couper

South Africa

Peter Alan Coventry

UK

Peter J Coventry

UK

Ernesto Crisafulli

Italy

Peter Croft

UK

Helen Croker

UK

Jesse Crosson

USA

Byron Crouse

USA

Walter Cullen

Ireland

Margaret Cupples

UK

Joanne Dalton

USA

Susan Darlow

USA

Rachel Davey

Australia

Darmon David

France

Guy de Backer

Belgium

Dinny de Bakker

Netherlands

Jan de Lepeleire

Belgium

Christian de Mey

Germany

Carl de Wet

UK

Hans Debruyne

Belgium

Jan M Degryse

Belgium

Ann Deketelaere

Belgium

Chris Del Mar

Australia

Eva Denison

Norway

Sarah Dennis

Australia

Siegrid Deutschlander

Canada

Myriam Deveugele

Belgium

Radhika Devraj

USA

Esperanza Diaz

Norway

Jon Dowell

UK

Frances Drummond

Ireland

Kristina Edvardsson

Sweden

Adrian Edwards

UK

Günther Egidi

Germany

Bernice Elger

Switzerland

Alison Elliott

UK

Jon Emery

Australia

Elizabeth Evans

UK

Andrew Farmer

UK

Julie Ferguson

UK

Nicholas Finer

UK

Yacov Fogelman

Israel

Yvonne Forsell

Sweden

Nicole Fowler

USA

Chester Fox

USA

Emma France

UK

Nick Francis

UK

George Freeman

UK

Jan C. Frich

Norway

Niels Frimodt-Moller

Denmark

Mark Gabbay

UK

Adel Gabriel

Canada

Rose Galvin

Ireland

Charmaine Gauci

Malta

Suad Ghaddar

USA

George Giannakopoulos

Greece

Hazel Gilbert

UK

John Gillies

UK

Julie Glanville

UK

Liam Glynn

Ireland

Dianne Goeman

Australia

Jaime Gofin

USA

J. Travis Gossey

USA

Merryn Gott

UK

Martine Granek-Catarivas

Israel

Liz Grant

UK

Barbara Griffin

Australia

Ole Rikard Haavet

Norway

Julie Hadley

UK

Walter E. Haefeli

Germany

Hanan Hamamy

Switzerland

Susan Hamilton

UK

Karin Hannes

Belgium

Johan Hansen

Netherlands

Rebecca Hansen

UK

Pia Hardelid

UK

Mark Harris

Australia

Peter Harvey

Australia

Richard Hays

Australia

Hilary Hearnshaw

UK

Stephanie Heinemann

Germany

Christoph Heintze

Germany

Virginia Hernandez Santiago

UK

Helen Herrman

Australia

Sherri Hinchey

USA

Oliver Hirsch

Germany

Harriet Hiscock

Australia

Falk Hoffmann

Germany

Knut Holtedahl

Norway

Rogier Hopstaken

Netherlands

Faline Howes

Australia

Claire Hulme

UK

Baruch Itzhak

Israel

Cathy Jackson

UK

Eyal Jacobson

Israel

Monika Janda

Australia

Juergen John

Germany

Sharon Johnston

Canada

Kate Jolly

UK

Martina Kadmon

Germany

Margaret Kay

Australia

Amy Keenum

USA

Diane Kelly

UK

Ngaire Kerse

New Zealand

Janko Kersnik

Slovenia

Masuma Akter Khanam

Bangladesh

Michael Kirby

UK

Darinka Klancar

Slovenia

Andrew Knight

Australia

Evangelos Kontopantelis

UK

Kurt Kroenke

USA

Madelon Kroneman

Netherlands

Peter Kropp

Germany

Ilkka Kunnamo

Finland

Deirdre Lane

UK

James Larcombe

UK

Celia Larson

USA

Christos Lionis

Greece

Cara Litvin

USA

Christin Loeffler

Germany

Andrew Long

UK

Karina Lovell

UK

Andrew Luck

Australia

Kathy Lucke

USA

Jeannette Lynch

UK

Sara Macdonald

UK

Donald Mack

USA

Mohsen Maddah

Iran

Antonio Madueno

Spain

Rosa Magallon-Botaya

Spain

Anthea Magarey

Australia

Kirsti Malterud

Norway

Douglas Mapel

USA

Stephen May

UK

John McKay

UK

Robert McKinley

UK

Gary McLean

UK

Alexandra McManus

Australia

Natalie Medlicott

New Zealand

Christa Meisinger

Germany

Liesbeth E. Meuwissen

Netherlands

Thorsten Meyer

Germany

Nele Michels

Belgium

Andreas Mielck

Germany

Anita Misra-Hebert

USA

Joe Moran

Ireland

Richard Morriss

UK

Gregory Moullec

Canada

U. A. Müller

Germany

Sarah-Anne Munoz

UK

Peter Murchie

UK

Helen Murphy

UK

Marion Murphy

Ireland

Lucio Naccarella

Australia

Bård Natvig

Norway

Felix Naughton

UK

Corina Naughton

Ireland

Janneke Noordman

Netherlands

Patrick O’Connor

USA

Thomas O’Dowd

Ireland

Anke Oenema

Netherlands

Ali Olyaei

USA

Eva Ouedraogo

Canada

David Paul

USA

Winifred Paulis

Netherlands

Teresa Pawlikowska

UK

Stephen Peckham

UK

Michael Pentzek

Germany

Lieve Peremans

Belgium

Rafael Perera

UK

Eliane Perrin

Switzerland

Davorina Petek

Slovenia

Mila Petrova

UK

Athena Philis-Tsimikas

USA

Christine Phillips

Australia

Ellen Piek

Netherlands

David Pierce

Australia

Hilary Pinnock

UK

Leon Piterman

Australia

Rebekah Pratt

USA

Terence Prendiville

USA

Joanne Protheroe

UK

David Rankin

UK

Heiner Raspe

Germany

Shaunta Ray

USA

Robert Reid

USA

Roy Remmen

Belgium

Gerry Richardson

UK

Matthew Ridd

UK

Dieter Riemann

Germany

Lewis Ritchie

UK

Edwin Rogers

USA

Helena Roig Carrera

Spain

Ellen Rosenberg

Canada

Wilhelmina Ruijs

Netherlands

Imre Rurik

Hungary

William Rush

USA

Bruno Rushforth

UK

Stefan Russmann

Switzerland

Luis Rustveld

USA

Elizeus Rutebemberwa

Angola

Guy Rutten

Netherlands

Andrew Ryan

USA

Teresa Sakraida

USA

Hiroshi Sakura

Japan

Anne-Laure Samson

France

Lena Sanci

Australia

Samantha Scallan

UK

Henk Schers

Netherlands

Jeffrey Schnipper

USA

Martin Schulz

Germany

Benjamin Schüz

Australia

David Seamark

UK

Antoni Serrano

Spain

Maninder Singh Setia

India

Chris Shaw

UK

Eric Shaw

USA

Sarah Shih

USA

Douglas Simkiss

UK

Greg Simon

USA

Shameran Slewa-younan

Australia

Paula Smailes

USA

Joel Smith

UK

Susan Smith

Ireland

Sandra Smith

USA

Wolfgang Spiegel

Austria

Andrew Stoddart

UK

Tim Stokes

UK

Daniel Strech

Germany

Patricia Sunaert

Belgium

Esther Suter

Canada

Jane Taggart

Australia

Ryan Tandjung

Switzerland

Athina Tatsioni

Greece

Roger Thomas

Canada

Carolyn Thorpe

USA

Hans Thulesius

Sweden

Stephanie Tierney

UK

Sarah Tonkin-Crine

UK

Ioanna Tsiligianni

Greece

Laurene Tumiel Berhalter

USA

Christy Turer

USA

Christine Urquhart

UK

Rodolfo Valdez

USA

Leonel Valdivia

Chile

Ann van den Bruel

UK

Hendrik van den Bussche

Germany

Bart Van den Eynden

Belgium

Marianne A.B. van der Sande

Netherlands

Liset van Dijk

Netherlands

Mieke L van Driel

Australia

Hein van Hout

Netherlands

Harm van Marwijk

Netherlands

Sandra Helena van Oostrom

Netherlands

Anne Loes van Staa

Netherlands

Sophie Vaux

France

Cindy Veenhof

Netherlands

Latha Velayudhan

UK

Pierre Verger

France

Theo Verheij

Netherlands

Etienne Vermeire

Belgium

Benjamin Vicente

Chile

Tara Vijayan

USA

Shlomo Vinker

Israel

Michael Von Korff

USA

Sina Waibel

Spain

Katherine Waite

USA

David Walker

UK

Anne Walsh

Australia

Julia Walters

Australia

Reginald Washington

USA

Karl Wegscheider

Germany

Johan Wens

Belgium

John Westfall

USA

David Whitford

Bahrain

Martin Wilkinson

UK

Sara Willems

Belgium

Andrew Wilson

UK

Jenifer Wogen

USA

Hilde Wøien

Norway

Fiona Wood

UK

Taixiang Wu

China

Hanne Würtzen

Denmark

Patrick Yachimski

USA

Htut Ye

Myanmar

Michael Yelland

Australia

Philippa Youl

Australia

Doris Young

Australia

Jacqui Young

Australia

Steven Zeliadt

USA

Yan Zhang

USA

Frans G. Zitman

Netherlands

Pascal Zufferey

Switzerland

Sandra Zwolsman

Netherlands

## Pre-publication history

The pre-publication history for this paper can be accessed here:

http://www.biomedcentral.com/1471-2296/14/18/prepub

